# Intravenous Thrombolysis for Minor Acute Ischemic Stroke

**DOI:** 10.1111/acem.70148

**Published:** 2025-09-04

**Authors:** Brit Long, Michael Gottlieb

**Affiliations:** ^1^ Department of Emergency Medicine University of Virginia School of Medicine Charlottesville Virginia USA; ^2^ Department of Emergency Medicine Rush University Medical Center Chicago Illinois USA

**Keywords:** acute ischemic stroke, disabling, neurology, NIHSS, nondisabling, thrombolysis, vascular

**Table:** 

NNT color recommendation	Black (Harm > Benefit)
Summary heading	Intravenous thrombolysis lowered functional independence and increased deaths at 90 days
Benefits in NNT	No one was helped
Benefits in percentages	No one was helped
Harms in NNT (NNH)	– 1 in 40 was harmed (lower chance of 90‐day functional independence) – 1 in 66 was harmed (death)
Harms in percentages	Intravenous thrombolysis compared to standard care: – 2.5% lower chance of 90‐day favorable functional independence – 1.5% increase in risk of death
Efficacy endpoints	Excellent recovery (modified Rankin scale 0–1), favorable outcome (modified Rankin scale 0–2)
Harm endpoints	Mortality within 3 months
Who was in the studies	3364 participants ≥ 18 years with minor ischemic stroke

## 1 Narrative

Acute ischemic stroke (AIS) is a significant cause of disability and mortality. Minor strokes, with a NIH Stroke Scale (NIHSS) ≤ 5, comprise over 50% of AIS [1, 2, 3], and up to one‐third of patients experience functional impairment within 90 days due to evolving or recurrent stroke [3, 4, 5, 6]. Guidelines therefore suggest intravenous thrombolysis for minor AIS with disabling symptoms [7, 8]. Here we summarize a systematic review assessing the safety and efficacy of intravenous thrombolysis added to standard care in patients with minor AIS [9].

The review and meta‐analysis data summarized here include 4 trials of 3364 adult participants with minor AIS randomized to standard care with or without thrombolysis [9]. While the review authors collected data from trials enrolling exclusively minor AIS patients, they also extracted and reported on some data examining minor AIS subgroups from trials that enrolled patients with a broad spectrum of AIS severities. For this summary, we focused on comparisons from trials enrolling only minor AIS patients. The review authors defined minor AIS as NIHSS ≤ 5, regardless of whether symptoms were considered disabling. The age of included patients ranged from 56 to 80 years, and thrombolytic drugs used in the trials included alteplase, tenecteplase, and pro‐urokinase.

The primary endpoint of interest was excellent outcome at 3 months, defined as a modified Rankin scale (mRS) score 0 to 1. Secondary endpoints included favorable outcome at 90 days (mRS score 0–2), mortality at 3 months, and recurrent stroke. Safety outcomes included intracranial hemorrhage. However, because we have focused on final neurologic status as measured by mRS, any clinical impact of intracranial hemorrhage is integrated into this final assessment. Therefore, the review does not report intracranial hemorrhage separately from mRS.

The systematic review reported no improvement in excellent recovery with thrombolysis (odds ratio [OR]: 0.85; 95% confidence interval [CI]: 0.7 to 1.03) [9]. Thrombolysis did, however, appear to *lower* the odds of 90‐day independence (OR: 0.7; 95% CI: 0.6 to 0.99; absolute risk difference [ARD] 2.5%; number‐needed‐to‐harm [NNH]: 40) and *increase* mortality (OR: 2.4; 95%: CI 1.2 to 4.7; ARD: 1.5%; NNH: 66). There was no difference in recurrent stroke. The results were unaffected or minimally affected by inclusion of subgroup data from trials enrolling patients with a variety of stroke severities [9].

## 2 Caveats

There are important limitations to this review [9]. First, the level of heterogeneity among the trials was high. The included RCTs had differing definitions of disabling symptoms, agents, time windows for treatment, routine care strategies, concomitant interventions, and follow‐up times. These differences may arguably challenge the validity of statistically pooling the results. Second, participants experiencing strokes presenting with isolated dysarthria, ataxia, facial weakness, sensory symptoms, and other isolated symptoms not captured by the NIHSS were not enrolled. Third, other advanced treatments like endovascular therapy were not evaluated [10]. While all RCTs were assessed as having low risk of bias, there were concerns regarding data completeness and baseline imbalances.

In summary, thrombolysis worsened 90‐day independence and increased mortality in trial participants with minor AIS. Thus, we have assigned a color of Black (Harm > Benefit).

Despite its widespread use, thrombolysis for ASI remains controversial [11], particularly after scrutiny of the NINDS and ECASS trials' data [12, 13]. This systematic review supports the need for further research through well‐designed large multicenter trials to assess its efficacy or identify specific populations that may benefit from it.

## Conflicts of Interest

The authors declare no conflicts of interest.

## Data Availability

The authors have nothing to report.

## References

[1] J. D. Steinmetz , K. M. Seeher , N. Schiess , et al., “Global, Regional, and National Burden of Disorders Affecting the Nervous System, 1990–2021: A Systematic Analysis for the Global Burden of Disease Study 2021,” Lancet Neurology 23, no. 4 (2024): 344–381. 38493795 10.1016/S1474-4422(24)00038-3 PMC10949203

[2] M. Reeves , J. Khoury , K. Alwell , et al., “Distribution of National Institutes of Health Stroke Scale in the Cincinnati/Northern Kentucky Stroke Study,” Stroke 44, no. 11 (2013): 3211–3213. 24003048 10.1161/STROKEAHA.113.002881 PMC4632977

[3] H. Saber , K. Khatibi , V. Szeder , et al., “Reperfusion Therapy Frequency and Outcomes in Mild Ischemic Stroke in the United States,” Stroke 51, no. 11 (2020): 3241–3249. 33081604 10.1161/STROKEAHA.120.030898

[4] E. E. Smith , G. C. Fonarow , M. J. Reeves , et al., “Outcomes in Mild or Rapidly Improving Stroke Not Treated With Intravenous Recombinant Tissue‐Type Plasminogen Activator: Findings From Get With the Guidelines‐Stroke,” Stroke 42, no. 11 (2011): 3110–3115. 21903949 10.1161/STROKEAHA.111.613208

[5] P. Khatri , M. R. Conaway , K. C. Johnston , and Acute Stroke Accurate Prediction Study ASAP Investigators , “Ninety‐Day Outcome Rates of a Prospective Cohort of Consecutive Patients With Mild Ischemic Stroke,” Stroke 43, no. 2 (2012): 560–562. 22052513 10.1161/STROKEAHA.110.593897 PMC3426999

[6] J. G. Romano , E. E. Smith , L. Liang , et al., “Outcomes in Mild Acute Ischemic Stroke Treated With Intravenous Thrombolysis: A Retrospective Analysis of the Get With the Guidelines‐Stroke Registry,” JAMA Neurology 72, no. 4 (2015): 423–431. 25642650 10.1001/jamaneurol.2014.4354

[7] W. J. Powers , A. A. Rabinstein , T. Ackerson , et al., “Guidelines for the Early Management of Patients With Acute Ischemic Stroke: 2019 Update to the 2018 Guidelines for the Early Management of Acute Ischemic Stroke: A Guideline for Healthcare Professionals From the American Heart Association/American Stroke Association,” Stroke 50, no. 12 (2019): e344–e418. 31662037 10.1161/STR.0000000000000211

[8] E. Berge , W. Whiteley , H. Audebert , et al., “European Stroke Organisation (ESO) Guidelines on Intravenous Thrombolysis for Acute Ischaemic Stroke,” European Stroke Journal 6, no. 1 (2021): I–LXII. 10.1177/2396987321989865 PMC7995316 33817340

[9] M. F. Doheim , T. N. Nguyen , Y. Xiong , et al., “Meta‐Analysis of Randomized Controlled Trials on IV Thrombolysis in Patients With Minor Acute Ischemic Stroke,” Neurology 105, no. 3 (2025): e213863. 40674672 10.1212/WNL.0000000000213863

[10] M. Gottlieb , J. N. Carlson , J. Westrick , and G. D. Peksa , “Endovascular Thrombectomy With Versus Without Intravenous Thrombolysis for Acute Ischaemic Stroke,” Cochrane Database of Systematic Reviews 4, no. 4 (2025): CD015721. 40271574 10.1002/14651858.CD015721.pub2 PMC12019923

[11] The NNT , “Tissue Plasminogen Activator (tPA) for Acute Ischemic Stroke,” accessed August 11, 2025, https://thennt.com/nnt/tissue‐plasminogen‐activator‐tpa‐acute‐ischemic‐stroke.

[12] R. Garg and S. Mickenautsch , “Risk of Selection Bias Assessment in the NINDS Rt‐PA Stroke Study,” BMC Medical Research Methodology 22, no. 1 (2022): 172. 35705913 10.1186/s12874-022-01651-4 PMC9202115

[13] B. S. Alper , G. Foster , L. Thabane , A. Rae‐Grant , M. Malone‐Moses , and E. Manheimer , “Thrombolysis With Alteplase 3‐4.5 Hours After Acute Ischaemic Stroke: Trial Reanalysis Adjusted for Baseline Imbalances,” BMJ Evidence‐Based Medicine 25, no. 5 (2020): 168–171. 10.1136/bmjebm-2020-111386 PMC7548536 32430395

